# Decellularization of fish tissues for tissue engineering and regenerative medicine applications

**DOI:** 10.1093/rb/rbae138

**Published:** 2024-11-28

**Authors:** Wenhui Chen, Mengshi Chen, Siyi Chen, Siran Wang, Zijin Huang, Lining Zhang, Jiaming Wu, Weijie Peng, Huaqiong Li, Feng Wen

**Affiliations:** Yuhuan People’s Hospital, Taizhou, Zhejiang 317600, China; Key Laboratory of Biomaterials and Biofabrication for Tissue Engineering, Gannan Medical University, Ganzhou, Jiangxi 341000, China; Zhejiang Top-Medical Medical Dressing Co. Ltd, Wenzhou, Zhejiang 325025, China; Zhejiang Engineering Research Centre for Tissue Repair Materials, Wenzhou Institute, University of Chinese Academy of Sciences, Wenzhou, Zhejiang 325001, China; Yuhuan People’s Hospital, Taizhou, Zhejiang 317600, China; Zhejiang Mariculture Research Institute, Wenzhou, Zhejiang 325005, China; Zhejiang Engineering Research Centre for Tissue Repair Materials, Wenzhou Institute, University of Chinese Academy of Sciences, Wenzhou, Zhejiang 325001, China; Key Laboratory of Biomaterials and Biofabrication for Tissue Engineering, Gannan Medical University, Ganzhou, Jiangxi 341000, China; Zhejiang Engineering Research Centre for Tissue Repair Materials, Wenzhou Institute, University of Chinese Academy of Sciences, Wenzhou, Zhejiang 325001, China; Zhejiang Top-Medical Medical Dressing Co. Ltd, Wenzhou, Zhejiang 325025, China; Zhejiang Engineering Research Centre for Tissue Repair Materials, Wenzhou Institute, University of Chinese Academy of Sciences, Wenzhou, Zhejiang 325001, China

**Keywords:** decellularization, tissue engineering, fish tissue, biocompatibility, bioactive material

## Abstract

Decellularization is the process of obtaining acellular tissues with low immunogenic cellular components from animals or plants while maximizing the retention of the native extracellular matrix structure, mechanical integrity and bioactivity. The decellularized tissue obtained through the tissue decellularization technique retains the structure and bioactive components of its native tissue; it not only exhibits comparatively strong mechanical properties, low immunogenicity and good biocompatibility but also stimulates *in situ* neovascularization at the implantation site and regulates the polarization process of recruited macrophages, thereby promoting the regeneration of damaged tissue. Consequently, many commercial products have been developed as promising therapeutic strategies for the treatment of different tissue defects and lesions, such as wounds, dura, bone and cartilage defects, nerve injuries, myocardial infarction, urethral strictures, corneal blindness and other orthopedic applications. Recently, there has been a growing interest in the decellularization of fish tissues because of the abundance of sources, less religious constraints and risks of zoonosis transmission between mammals. In this review, we provide a complete overview of the state-of-the-art decellularization of fish tissues, including the organs and methods used to prepare acellular tissues. We enumerated common decellularized fish tissues from various fish organs, such as skin, scale, bladder, cartilage, heart and brain, and elaborated their different processing methods and tissue engineering applications. Furthermore, we presented the perspectives of (i) the future development direction of fish tissue decellularization technology, (ii) expanding the sources of decellularized tissue and (iii) innovating decellularized tissue bio-inks for 3D bioprinting to unleash the great potential of decellularized tissue in tissue engineering and regenerative medicine applications.

## Graphical abstract

**Figure:**
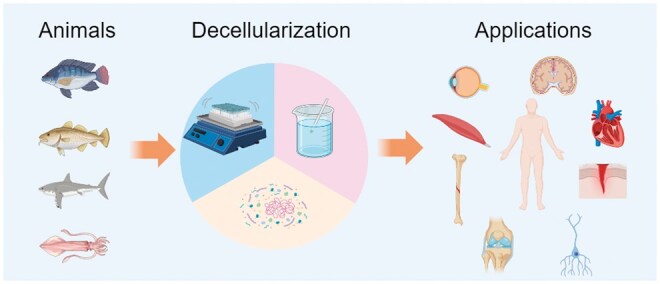

## Introduction

The extracellular matrix (ECM) is an assembly of a 3D network formed by various macromolecular substances secreted from cells and consists of various structural and functional proteins such as collagen, proteoglycan/glycosaminoglycan (GAG), elastin, fibronectin, laminin and other glycoproteins [1–3]. Matrix molecules bind to each other, and cell adhesion receptors fill the spaces between the cells and their junctions to provide 3D structural support for the surrounding cells to anchor. ECM regulates cell-inhabited behavior by establishing a microenvironment surrounding the cell [4]. It has a significant impact on cell behaviors, including adhesion, proliferation, migration, differentiation and functional expression, due to its tissue-specific morphological features as well as biochemical cues [5, 6]. Therefore, they play a vital role in tissue development, homeostasis and disease progression. Tissue engineering offers a promising strategy to restore damaged tissues and consists of three main components: cells, signaling molecules and scaffold [7–9]. Scaffolds made from ECM are attracting intensive attention because of their native structures, comparatively high bioactivity and tunable biodegradability, which are difficult to imitate using synthetic materials [10]. However, the insufficient autologous ECM has restricted its application. Since 3000 BC, ancient Egyptians have used xenogeneic ECM as medical device-wound suture derived from linen thread, which have been found in mummified remains. Nevertheless, due to cellular components remnants in ECM, such as deoxyribonucleic acid (DNA), galactose-alpha-1,3-galactose (α-Gal) epitopes and major histocompatibility complex I, which are recognized as foreign substances by the host, and immune-mediated rejections induced subsequently. Therefore, their tissue engineering applications as scaffolds are tremendously limited [11–13].

**Figure 1.**
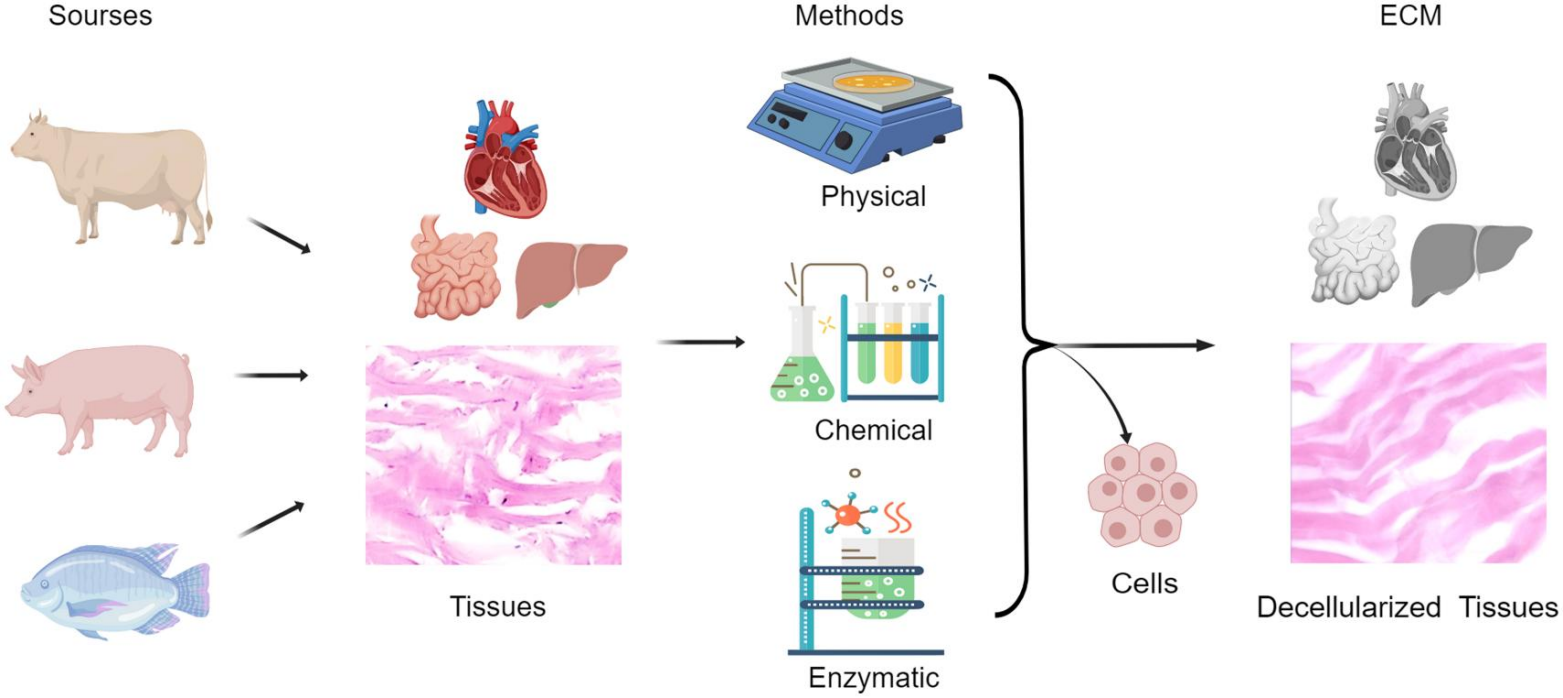
Flowchart of the decellularization process. It illustrates how tissues from different sources are decellularized using different methods to produce the decellularized tissue. Created with MedPeer (medpeer.cn).

**Figure 2.**
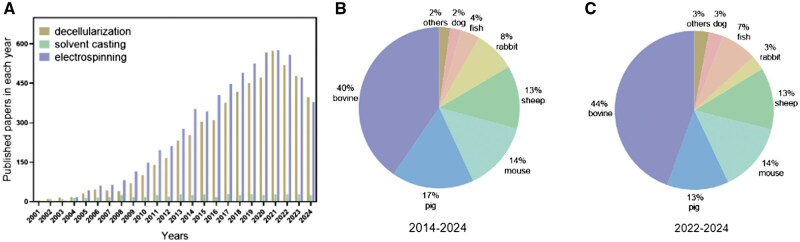
Status of decellularization research in the tissue engineering field. (**A**) The numbers of publications worldwide on decellularization research in the tissue engineering field found in PubMed. (**B**) and (**C**) Scales of the various animals used for decellularization, which were searched from PubMed. Date of search: October 21, 2024.

Decellularization is the process of obtaining acellular tissues with low immunogenic cellular components from animals or plants while maximizing the retention of the native ECM structure, mechanical integrity and bioactivity. It removes immunogenic cellular residues associated with the tissue to reduce the host immune response, thereby enhancing the biocompatibility of acellular tissues [10, 14]. Tissue consists of cells and ECM, and the major component of tissue is mostly ECM. Decellularized tissue is also referred to as acellular tissue or ECM. Figure 1 illustrates the process of tissue decellularization, in which cells in bovine, porcine and fish tissues are removed using physical, chemical and enzymatic methods, leaving only the ECM. The section on the left shows the tissues harvested from three types of animals: bovine, pig and fish. The middle section shows that the tissues were decellularized using physical, chemical and enzymatic methods. The right section shows decellularized tissues-ECM. The challenge of decellularization is to balance the demands between minimum residual immunogenic cellular components in the ECM and the preservation of the structural and mechanical integrity and biological activity of the ECM. Decellularization is becoming a widely recognized methodology for fabricating scaffolds consisting of ECM and is gaining momentum in the tissue engineering research field [15–17]. Figure 2A shows the numbers of publications worldwide on decellularization research in the tissue engineering field found in PubMed. The papers including the key words of ‘decellularization’ and ‘tissue engineering’ in All Fields were counted while the papers including ‘solvent casting’ or ‘electrospinning’ were counted as controls. Solvent casting is a popular methodology, and electrospinning is one of the most frequently used methods for fabricating scaffolds in the tissue engineering research field [18]. As shown in Figure 2A, a large number of publications on decellularization in recent years have been published. Similar to electrospinning, research on decellularization has also been receiving intensive attention. At the same time, the scales of the various animals used for decellularization searched from PubMed for the last 10 and 3 years are shown in Figure 2B and C. ‘Decellularization’ and each animal name (such as ‘decellularization and pig’ or ‘decellularization and fish’ in the title/abstract) were used as keywords for this search. As shown in the two figures, tissues from bovine, pig, mouse, sheep, rabbit and fish have been the top six animal tissues used for decellularization research over the past 10 and 3 years. However, the share of fish tissue as a source of decellularization material has increased from 4% in the past 10 years to 7% in the past 3 years, implying that fish tissue decellularization has recently gained more attention. Poel *et al.* [19] started the first exploration of decellularization for the preparation of acellular homogenates from muscle samples in 1948. Since then, more decellularization technologies have been explored, and various acellular tissues have been produced and applied in a broad range of tissue engineering applications, such as dermal tissue repair [20–22], bone tissue repair [23, 24] and heart regeneration [25, 26]. Currently, most commercial acellular tissue products are decellularized from human, bovine or pig origins [27]. For example, AlloDerm^®^ (Lifecell), decellularized from the human dermis, CollaMend FM Implant (C.R. Bard) decellularized from porcine dermis, Oasis^®^ (HealthPoint) decellularized from porcine small intestinal submucosa and Dura-Gaurd^®^ (Synovis Surgical) decellularized from bovine pericardium. Moreover, through post-processing of decellularization technology, the physical structural transformation of acellular tissue (hydrogel, powder) facilitates its application in different scenarios according to clinical needs, and can also be used to prepare bio-ink materials for 3D bioprinting, greatly expanding the clinical application range of acellular tissue and enhancing its economic value [28].

Recently, there has been a growing interest in the decellularization of fish tissues to avoid religious constraints and the risk of zoonotic pathogen spillovers, such as hand–foot–mouth disease, bovine spongiform encephalopathy and swine influenza. Most fish diseases are specific to fish and pose no threat to humans, with a few exceptions, such as Mycobacterium marinum infection and Ichthyophthirius multifiliis caused by bacteria, parasites, viruses and fungi, which are similar to diseases in other animals [29]. However, most of the identified natural reservoirs of zoonotic pathogens are mammals (∼80%), followed by avian [30]. In addition, decellularization often involves treatments such as chemical or physical agents, which can destroy or remove many pathogens hosted in the tissue. This will significantly reduce the risk of pathogen transmission when decellularized tissue is used in tissue engineering and regenerative medicine. In addition to the mitigation of pathogen transmission, fish and human tissue ECM share some similarities in terms of physical structure (complex 3D framework), biological composition (collagens, laminins, fibronectin, GAGs and proteoglycans) and physiological functions (regulating tissue development and homeostasis) [31]. However, there are also significant differences (the specific types and ratios of ECM components and mechanical properties). Currently, most studies on fish tissue decellularization have focused on the skin [32–39], scale [40–44], bladder [45–47], brain [48] and heart [25]. These decellularized tissues have been used as biomaterials to improve regeneration or replacement of site-appropriate tissues *in vivo*. Here, we provide a complete overview of the state-of-the-art decellularization of fish tissue on organs and methods for preparing acellular tissue. We have also updated the tissue engineering applications of acellular tissue derived from different tissues and organs in fish. In addition, we present the prospects for the future development of fish decellularization research and its potential in tissue engineering applications.

## Methodology for decellularization

The goal of decellularization is to remove cellular antigens associated with the native ECM that triggers an immune response *in vivo*, while preserving the intact ECM composed of native structural and functional molecules [49]. The properties of the decellularized tissue can be affected by different decellularization methods. Various decellularization methods have been developed and can be divided into three categories: physical, chemical and enzymatic methods [50]. The optimal decellularization method is to completely remove the cellular components while retaining the original structure and components, giving it the same biochemical and mechanical properties as the native ECM. Ideally, decellularized tissues replicate the complexity of the native microenvironment to achieve optimal regenerative capacity and functional recovery.

### Physical methods

The physical method obtains acellular tissue by destroying the cell membrane and removing cell components by regulating the physical characteristics of the tissue (such as temperature, force and pressure). The most significant advantage of this treatment is that there are no residual chemicals or enzymes; thus, there is no cytotoxicity in decellularized tissue. The most significant disadvantage of this method is its inadequate decellularization efficiency when used alone. Physical methods of decellularization include freeze-thaw cycles, immersion and agitation.

#### Freeze-thaw cycle

The freeze-thaw cycle, which is often used as an aid for decellularization, is the most common decellularization method. By rapidly freezing water in tissues, intracellular ice crystals can subsequently puncture the cell membranes and cause cell lysis [25, 32]. However, enlarged ice crystals may also destroy the ultrastructure of the ECM, so cryoprotectants such as trehalose and dimethyl sulfoxide are usually added to control the size of ice crystals [51]. Because the freeze-thaw cycle has less effect on mechanical properties, this method is generally preferred for acellular tissues with strong bearing capacity and high mechanical strength requirements. Many recent studies have prioritized the decellularization of tissues by freeze-thaw cycles to initiate cell membrane destruction, followed by the use of detergents such as Triton X-100 and sodium dodecyl sulfate (SDS) to remove leaked cell contents. The decellularization efficiency of this method depends mainly on the cooling/thawing rate, temperature difference, treatment time and cycle number. There is no exception to fish tissue decellularization. Chen *et al.* [35] froze black carp skins in an environment of −40°C, and then thawed it with tap water at room temperature and then the skins were stirred in 1% Triton X-100 for 12 h and 0.5 μg/ml trypsin (pH 8.0 Tris–HCl buffer) for 18 h for further decellularization. The results showed that the acellular tissue had both good biocompatibility and high mechanical properties and promoted angiogenesis and collagen synthesis in the rat skin wound area. However, there was no quantitative detection of residual chemicals and DNA in the decellularized tissue in this study; only a qualitative analysis of the cell nucleus was conducted using staining.

#### Immersion and agitation

As with the freeze-thaw cycle, immersion and agitation are often used as aids in tissue decellularization [22, 25]. Some thick tissue structures have fewer blood vessels, making it difficult for the decellularization solution to enter the center of the tissue, so other decellularization strategies are needed to assist such tissues. The most common and simplest method is to immerse the tissue in a decellularization agent while stirring to accelerate the decellularization agent into the center of the tissue. Immersion and agitation could improve mass transfer to deliver the decellularization solution to all parts of the tissue. This method has been adopted for a variety of tissues, including heart valves [52], blood vessels [53], skeletal muscles/tendons [54], spinal cord [55], tracheal [56], dermis [57] etc. The duration of decellularization using this method is related to the tissue thickness, density, type of decellularization solution and agitation intensity. Very thin tissues, such as the submucosa of the small intestine and urinary bladder, can be effectively decellularized after immersion in peracetic acid with agitation for a relatively short period of time. Remya *et al.* [58] developed an acellular tissue by decellularization of fish swim bladder (FSB) for wound repair. FSBs were decellularized using 0.5% sodium deoxycholate for 24 h. To achieve better contact between the tissue and decellularization solution, the FSBs were immersed in the decellularization solution with continuous agitation at a rate of 180 revolutions/min in a horizontal orbital shaker at 37°C during the decellularization. The results demonstrated the complete removal of cellular components and mild derangement of the FSB architecture, providing adequate porosity for cell proliferation.

### Chemical methods

Chemical methods include the use of detergents, bases, acids, hypotonic and hypertonic solutions, alcohol and other solvents to disrupt cell membranes and junctions, remove cellular components and ultimately obtain acellular tissue. The most prominent advantages of this decellularization method are high efficiency and thoroughness. However, if subsequent cleaning is not thorough, a small amount of residual chemicals may remain in the ECM, thus introducing cytotoxicity to the decellularized tissue.

#### Acids and bases

Acids and bases have also been used as fish tissue decellularization agents [22, 32, 41]. They can denature proteins, dissolve cell components, change nucleic acids and cause cell rupture [59]. Acids dissociate DNA from the ECM by solubilizing cytoplasmic components and disrupting nucleic acids. Acetic acid did not affect the protein composition and GAG in the ECM but could weaken the strength of the ECM through damage to collagen composition. Bases are more destructive to tissues, can destroy growth factors and disrupt the cross-linking of collagen fibers, thus weakening the mechanical properties of acellular tissues. Wang *et al.* [60] reported a decellularization method for Basa fish skin including bases treatment combined with freeze-thaw cycle for wound repair application. Fish skins were soaked in 4% sodium hydroxide (NaOH) for 18 h to remove fat from the skin and then subjected to three cycles of freeze-thaw for decellularization. Blood compatibility and systemic acute toxicity examination results showed that the acellular tissue had a hemolysis rate of less than 5% and no acute systemic toxicity. Besides decellularization, acid was also used to increase the porosity of the decellularized tissue and decalcified the fish scales to improve the biocompatibility of the acellular tissue [44].

#### Non-ionic and ionic detergents

Detergents are essential for tissue decellularization. Many studies on acellular fish tissues have used non-ionic detergents in the decellularization process [35, 42]. Compared with ionic detergents, after treatment with non-ionic detergents, the functional conformation of proteins in the ECM is well preserved. Triton X-100 is one of the most widely used non-ionic detergents. Accompanied by the loss of ECM proteins, it can remove cellular residues to mitigate adverse immune reactions in the host. However, whether it can successfully achieve the effective removal of cellular components in the ECM depends on the composition and structure of the source tissue. Li *et al.* [22] developed a novel tilapia skin acellular tissue to prompt wound healing. During the decellularization process, fish skins were first soaked in 0.1% Triton X-100 (w/w) at 8°C for 16 h, and then immersed in a 0.1-M NaOH solution at 8°C for 6 h. All the above steps were performed under agitation at 50 rpm. In the hematoxylin and eosin (H&E) and 4′,6-diamidino-2-phenylindole (DAPI) staining images, residual nuclei in the acellular tissue were absent. In addition, through quantitative measurement, the residual DNA content in acellular tissue was 1.4 ± 0.7 ng/mg, which was far below the limit recognized by the medical industry [2, 6, 61].

Ionic detergents such as SDS and sodium deoxycholate are known to disrupt cell membranes and denature proteins [1, 62]. SDS is a powerful cleanser that removes most of the components in tissues, except collagen. In addition, because SDS can disrupt the interactions between cells and between cells and the ECM, disrupt protein–protein interactions and emulsify phospholipids, it can effectively remove cellular components from the tissues [63]. Although SDS can effectively remove immunogenic cellular components from tissues, it may also damage ECM structures and signaling proteins. The decellularization effect of SDS is related to treatment duration and its working concentration. The most common SDS decellularization concentration is 0.1% [64]. Liu *et al.* [46] reported a novel biomaterial-acellular FSB tissues from hypophthalmichthys molitrix for cardiovascular defect repair. FSB tissues were firstly soaked in 1% (w/v) SDS and 1% (v/v) Triton X-100 under agitation at room temperature. Subsequently, they were immersed in deoxyribonuclease (DNase) and ribonuclease (RNase) overnight at 37°C under agitation. The results showed that, after decellularization, most of the cellular components in the FSB acellular tissue were removed. DNA quantitative analysis showed that the DNA content in acellular tissue is 0.01 ± 0.01 μg/mg, much less than that in bovine pericardium acellular tissue (0.11 ± 0.03 μg/mg), thus largely mitigate its potential immunogenicity *in vivo*. However, it should be noted that residual chemical reagents must be carefully removed after decellularization. Even a small amount of reagent residue may cause severe cytotoxicity and negate the advantages of decellularized tissue as a bioactive material.

### Enzymatic methods

The enzymes available for tissue decellularization include nuclease, trypsin, lipase, dispase, thermolysin and α-galactosidase [1, 10, 65]. Enzymes can specifically remove cell debris and ECM components. However, it is difficult to completely remove cellular components in the tissue alone, and the residual enzymes in the acellular tissue can destroy the recellularization of acellular tissue and stimulate a serious immune response. Nucleases include DNase and RNase, which hydrolyze deoxyribonucleotide and ribonucleotide chains, respectively. Therefore, they degrade nucleic acids and enable their rapid removal after cell lysis. Additionally, nucleases can affect the composition of ECM components, thereby influencing cell proliferation during the recellularization process [61]. Trypsin is also commonly used for decellularization. It cleaves peptides at the C-termini of lysine and arginine residues [1]. Proteins in the ECM, such as collagen, can also be digested using trypsin. Compared with detergents, the use of trypsin to remove cells is slower. It is typically used to enhance tissue permeability and facilitate the penetration of subsequent decellularization agents. Lipase can act on specific sites on the glycerol backbone of lipid substrates, helping to remove lipids from the ECM and being suitable for binding with lipid coenzymes to enhance its efficiency. Dispase is a neutral protease that breaks down fibronectin and type IV collagen. It has been used for decellularization of the cornea and skin [14]. The α-Gal epitope is present on the cell surfaces of non-primate mammals, marsupials and New World monkeys but not on human cell surfaces [66]. The binding of natural antibodies on human cell surfaces with the α-Gal antigen on implants leads to hyperacute rejection in xenotransplantation [66, 67]. α-Galactosidase is a glycoside hydrolase that catalyzes the hydrolysis of α-galactosidic bonds and can be used to reduce surface antigens (α-Gal epitopes) in acellular tissues. Thermolysin is a zinc-containing enzyme that hydrolyzes proteins into smaller peptides and/or amino acids by cleaving the peptide bonds. However, it is only effective during the removal of cells from the surface of the tissues. The combined application of dispersing enzymes and detergents can further improve the degree of tissue decellularization. Chen *et al.* [35] developed an acellular skin from mylopharyngodon piceus to promote wound healing. They first soaked the fish skin in 1% Triton X-100 with stirring for 12 h, then soaked it in 0.5 µg/ml trypsin for 18 h to decellularize the fish skin. Histological photographs of the decellularized tissue revealed the porous structure of the fish skin acellular tissue. Lau *et al.* [33] firstly soaked tilapia skin in dispersing enzyme solution, and then immersed in 1% SDS, to lysed cells, and released cell contents. Finally, the nucleic acid is degraded by nuclease. After decellularization, the DNA and collagen content in tilapia skin decreased from 419.9 ± 109.5 ng/mg and 522.9 ± 45.0 μg/mg to 1.8 ± 0.9 ng/mg and 362.5 ± 35.6 μg/mg, respectively, indicating that the decellularization using combination methods significantly reduced its potential immunogenicity while retaining most of the bioactive components and the microstructure of the natural ECM.

Decellularization begins with the dissolution of cell membranes by physical or chemical means, followed by the separation and removal of cellular components from the ECM by enzymatic treatments and detergents [51]. Lin *et al.* [66] reported a decellularization method to prepare acellular tissue from tilapias scales to maintain sufficient mechanical property for corneal regenerative applications. This method included a four-step extraction process. The scales were firstly soaked in a hypotonic Tris-buffer containing a protease inhibitor for 24 h with continuous stirring. Next, they were immersed in 1% Triton X-100 in Tris-buffer with protease inhibition for 24 h with continuous stirring. In the third step, DNase and RNase digestion was performed at 37°C for 1 h. Finally, extraction with Triton X-100 in Tris-buffer for 24 h. Scanning electron microscopy (SEM) images showed that the decellularization process did not damage the natural 3D, highly centrally oriented microstructure of the native ECM. To examine the cytocompatibility of the acellular tissue of scales, rabbit corneal cells were cultured on acellular tissue, and cell morphologies were assessed by SEM and confocal microscopy at different time points. The results demonstrated the superior cellular conductivity of the acellular tissue and provided convincing evidence for the feasibility of decellularized scales as a promising biomaterial for corneal tissue engineering. However, there are no data illustrating the residual DNA in the acellular tissue, which is critical to overcome super acute transplantation immune rejection and has better clinical therapeutic effects. The current standards for decellularized tissue include: (i) <50 ng DNA/mg acellular tissue (dry weight), (ii) <200 bp DNA fragment length and (iii) lack of visible nuclear materials in tissue sections stained with DAPI or H&E [68]. Complete decellularization of any tissue using a single chemical or physical method is often difficult due to the limitation of DNA removal efficiency of a single method, while other properties of acellular tissue such as mechanical strength and biological composition (collagen, GAG, etc) need to be preserved as well. Residual DNA and other cytoplasmic and nuclear materials in acellular tissue can trigger problematic immune responses, such as chronic inflammation, when in excess after use *in vivo*. Therefore, current methods for decellularizing fish tissues often involve a combination of two or three of the physical, chemical and enzymatic methods [69, 70] as shown in Table 1 [27, 29]. The major challenge and pursuit in the development of decellularization technology is to achieve an acellular tissue free of chemical and immunogenic cellular residues, while preserving the original structure and mechanical properties of the natural ECM.

**Table 1.** Methods of decellularization for fish tissues

Methods		Mechanism	Advantages	Disadvantages	References
Physical	Freeze-thaw cycle Immersion and agitation Electrophoresis	Form intracellular ice crystals, disrupt cellular membrane Improve mass transfer of decellularization solution Improve cell membrane permeability	Maintain ECM protein Convenient-Low toxicity	Inadequate decellularization efficiency Damage the ultrastructure of ECM High temperature	[25, 32, 41, 68–72] [22, 25, 32, 33, 35, 41, 42, 44, 46, 68, 73–81] [69]
Chemical	Ionic and non-ionic detergents	Solubilize cell and nucleic membranes, denature proteins Disrupt ECM structure and remove desirable biological molecules (e.g. GAG, growth factors, etc.)	Highly effectively remove cellular components	Damage ECM structure integrity Reducing specific components and bioactivity of ECM High toxicity of residual detergent	[22, 28, 32, 33, 35, 41, 42, 44, 46, 47, 68, 70, 71, 73–75, 78–89]
	Acid and base	Solubilize cytoplasmic components of cells Degrade genetic material Denature protein	Effectively remove cellular and nuclear components	Reduce some growth factors and decrease the bioactivity of ECM Damage ECM structure and reduce GAGs	[22, 32, 41, 73]
Enzymatic	Nuclease Dispase and trypsin	Catalyze the hydrolysis of RNA and DNA Cleavage of various peptide bonds	Precisely remove cellular components	Long processing time Damage the ECM structure Promote immune response	[33, 42, 44, 46, 69, 72, 87, 89] [33] [32, 35, 68, 70, 84, 88]

## Fish tissues for decellularizations and applications

**Figure 3.**
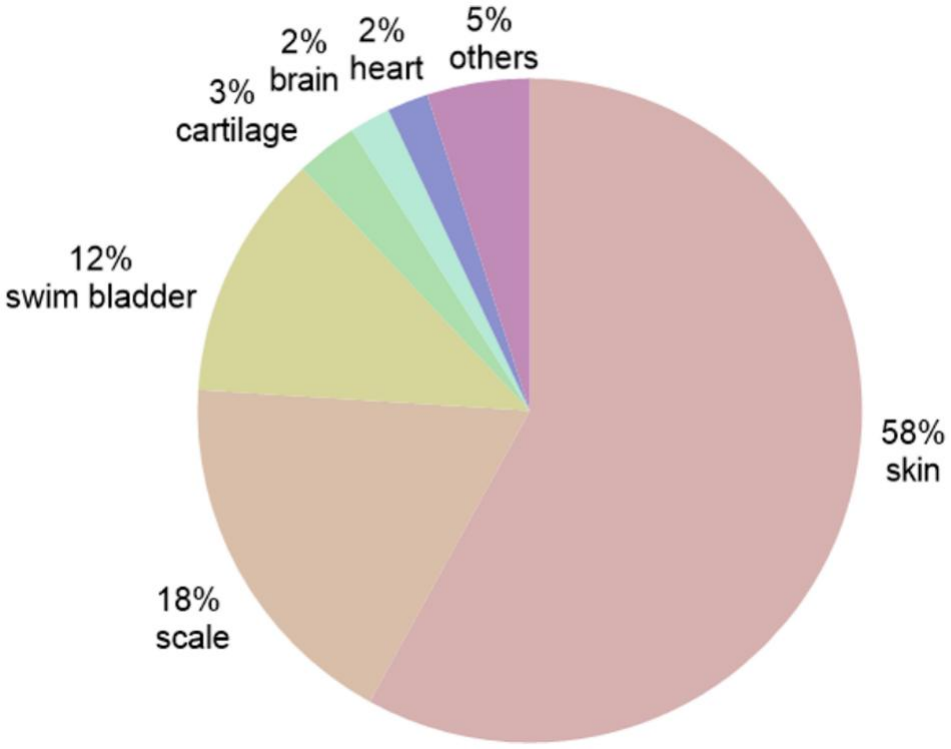
Pie chart for the relative percentage of studies reported on various types of fish acellular tissues derived from different tissues over the last 10 years. Date of search: October 21, 2024.

World fish production was 177.8 million tons in 2019 and was expected to continue to grow in the future. Approximately 70% of fish production must be processed before consumption [90]. Fish processing (beheading, de-shelling, degutting, removal of fins and scales and filleting) produces many by-products [91]. These by-products are usually composed of viscera, cut parts, heads, fins, skins, scales and bones, accounting for ∼50–75% of fresh weight, depending on the species [92]. Between 50% and 70% of by-products are considered ‘inedible’ and usually consist of trimming [93]. Landfills or incineration results in environmental, health and economic damage [94]. However, these by-products have potential biomedical applications. After decellularization, fish tissues exhibit good biocompatibility, biodegradability and bioactivity, promoting their applications in tissue engineering and regenerative medicine [25, 33, 40, 46, 81, 95]. The development and application of decellularization technology in fish tissues not only provide a large amount of high-quality biomaterials for medical use and improve our living environment but also eliminate the large amount of waste generated in the fishing industry. In line with recent highlights of decellularization of fish tissues, we enumerated the common decellularized fish tissues from various fish organs, such as skin, scale, bladder, cartilage, heart and brain, and elaborated their different processing methods and their tissue engineering applications (Figure 3 and Table 2). Many publications worldwide on fish tissue decellularization research have been found in PubMed. The papers including the key words of ‘decellularization’ and ‘fish’ in All Fields in last 10 years were counted. Subsequently, by reviewing their experimental methods, we categorized the studies by tissue.

**Table 2.** Applications of fish tissues decellularized using different methods

Fish tissues	Decellularization methods	References	Applications	References
Skin	Chemical treatment Enzymatic treatment Physical treatment	[22, 32–37, 39, 60, 68, 73, 75–78, 85, 88, 95, 98–106] [32, 33, 35, 88] [32, 68, 76, 77]	Wound healing Dura repair Ulcer healing Bone repair Muscle repair	[22, 34–37, 39, 60, 73, 75, 77, 78, 85, 88, 98, 100–106] [99] [95] [32, 33, 73] [68]
Scale	Chemical treatment Enzymatic treatment	[40, 43, 44, 82, 89, 107–109] [43, 89, 107–109]	Corneal regeneration Bone repair	[43, 44, 89, 107–109] [40, 82]
Swim bladder	Chemical treatment Physical treatment Enzymatic treatment	[45–47, 69, 110] [45, 71, 110] [46, 69]	Dura repair Wound healing Cardiovascular materials	[47, 69] [71, 110] [45, 46]
Cartilage	Chemical treatment Enzymatic treatment Physical treatment	[81, 87] [81, 87] [87]	Cartilage repair	[81, 87]
Heart	Enzymatic treatment Physical treatment	[25] [25]	Cardiovascular materials	[25]
Brain	Enzymatic treatment Physical treatment	[72] [72]	Nervous system repair	[72]

Tilapia is a tropical freshwater fish that is becoming a promising source of fish collagen because of its popularity on fish farms, rapid growth and high protein content. In addition, tilapia skin with thin-film geometry, mainly composed of type I collagen, has been proven to have good biocompatibility [33, 68], low immunogenicity and wound-healing promoting activity [22]. Therefore, it has been adopted in clinical practice [96, 97]. Li *et al.* [22] prepared a low-cost bioactive wound dressing from tilapia skin acellular tissue, using base and Triton X -100 as decellularization solution. This bioactive dressing significantly promoted granulation growth, collagen deposition, angiogenesis and re-epithelialization in the porcine full-thickness skin defect area due to appropriate degradation of the acellular tissue and the conducive microenvironment established for wound healing.

The Atlantic cod fish skin acellular tissue named Kerecis Omega3 wound has been approved for wound repair by the U.S. Food and Drug Administration. Kerecis Omega3 wound has similar macro- and microstructure to human skin, which means the acellular tissue naturally presents ideal pore size and geometry for the ingrowth and attachment of human skin cells [37, 98]. This acellular tissue retains most of its native bioactive compounds, including type I collagen and high content of omega-3-fatty acids. The biological property of omega-3-fatty acids is a hot topic of research [36, 99, 100], and its contributions to increased cell migration rates, anti-inflammatory, analgesic and antimicrobial [101] effects in accelerating wound healing have been demonstrated. Another research hotspot is the comparison of acellular tissue with other prevailing skin substitutes in the market for different clinical applications, which appears to provide promise as a safe dura patch [99] and effective wound coverage for acute wounds [34, 98, 105], hard-to-heal ulcers [95], diabetic lower extremity wounds [35, 38, 102], burn wounds [106] and battlefield trauma [101]. Although the physical and chemical properties of fish skin (basa, black carp, grass carp, leather jacket fish, etc) are different, they can still be decellularized using individual methods and cross-linked to achieve ideal mechanical properties for various applications [76]. Wang *et al.* [32] investigated the potential use of an acellular tissue of astroconger myriaster skin as a guided tissue regeneration membrane in oral medical applications. The results showed that acellular tissue from the skin of the astroconger myriaster possessed good biocompatibility and very low cytotoxicity.

**Figure 4.**
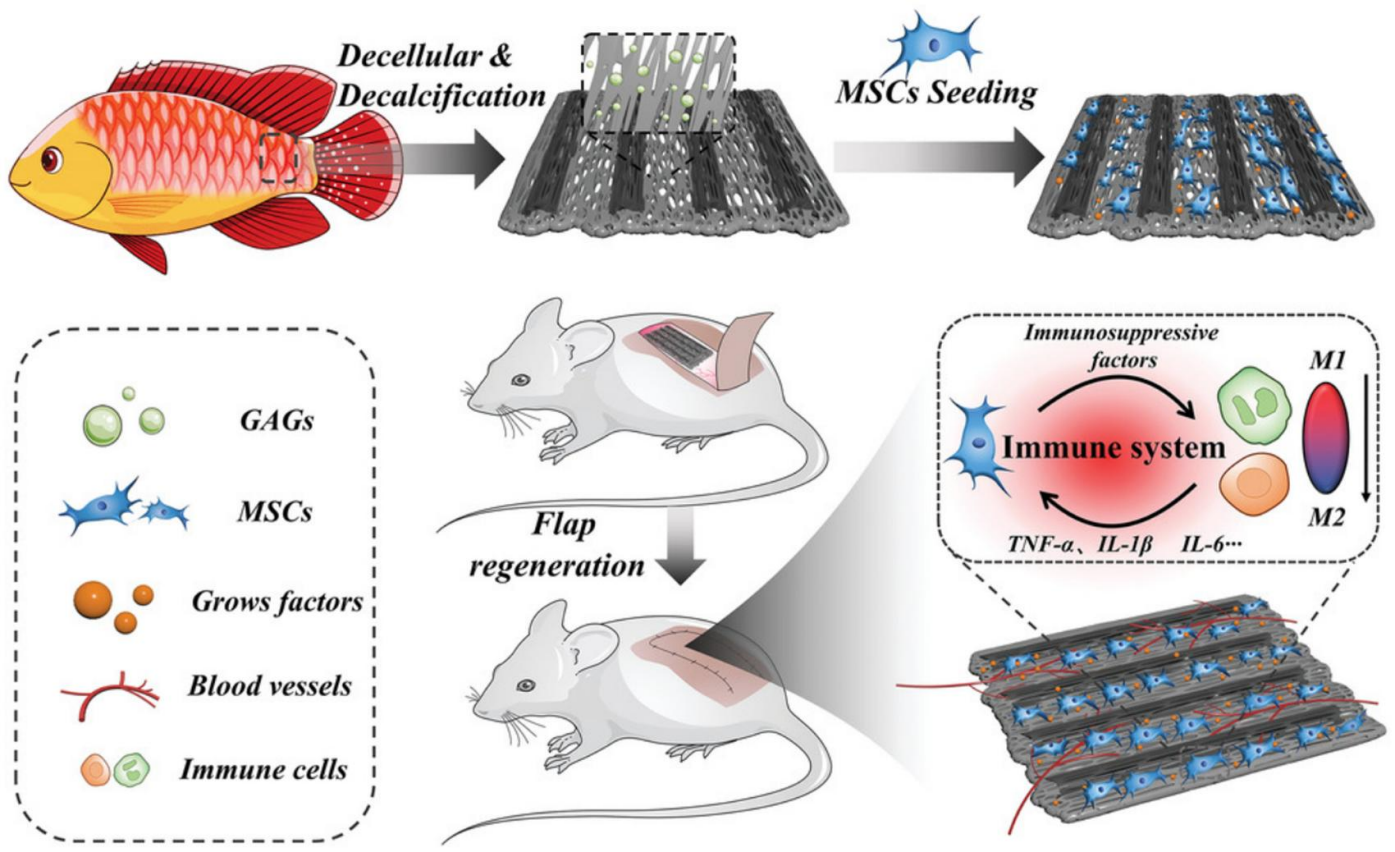
Schematic diagram of the process of promoting flap survival using MSC attachment scaffolds prepared from decellularized and demineralized fish scales. Reproduced from Ref. [41] with permission from John Wiley and Sons @ 2024.

Fish scale is a biodegradable and low-cost biological source. Currently, the industry is mainly focused on the extraction of type I collagen and hydroxyapatite (HAp), which are the main components of fish scale. Nevertheless, the distinct organization structure of the teleost elasmoid scale has caught the attention of scientists in the last decade because of its excellent mechanical properties. The 3D structure of the teleost elasmoid scale is highly ordered and composed of two layers. Collagen fibers are randomly arranged in the outer layer, while collagen fibers are organized into lamellae to form an orthogonal plywood-like pattern in the fibrillary internal layer. The consecutive fibers construct microchannels, and needle-shaped HAp crystals are distributed in the ordered arrangement of collagen fibers in the inner layer. Because of the physical and chemical similarities between teleost elasmoid scales and bones, acellular tissue from teleost elasmoid scales has been used to produce bioabsorbable bone pins. Chou *et al.* [40] developed a novel bone pin using decellularized grass carp scales. Decellularization was performed in 0.05 M Tris-buffer and 0.1 M Triton X-100 solution. The characterization results indicated that the bone pin had good mechanical properties (Young’s modulus: 332 ± 50.4 MPa; tensile strength: 34.4 ± 6.9 MPa). In a rabbit femoral fracture model, bone pins showed good biocompatibility and degradation performance. Kara *et al.* [82] used acellular fish-scale microparticles as a reinforcement and chitosan to fabricate a novel 3D porous composite scaffold for bone tissue regeneration. Acellular fish-scale microparticles increased the viability and proliferation of SaOS-2 cells in scaffolds owing to their high collagen content. Collagen and HAp in the fish-scale also promote alkaline phosphatase activity, osteocalcin secretion and biomineralization, which are major markers of osteogenic differentiation. Lin *et al.* [41] prepared a novel biohybrid tissue construct by integrating the advantages of fish-scale acellular tissue and stem cells for skin flap regeneration as shown in Figure 4. Through the decellularization and decalcification processes, the microchannel morphology of the fish scale was well preserved, which could provide an anisotropic template for the oriented growth of cells. In a rat flap model study, biohybrid tissue constructs significantly reduced the area of necrosis in the distal flap, effectively promoted angiogenesis and macrophage M2 polarization, and increased the survival rate of the skin flap.

The unique arrangement of collagen fibers in the teleost elasmoid scale also resembles that of the corneal stroma. After decellularization and decalcification, tilapia fish scales show excellent light transparency and scattering [89]. In addition, fish-scale acellular tissue is permeable to gas [107] and glucose [108], which are prerequisites for artificial corneal implants. Moreover, fish-scale acellular tissues have excellent biocompatibility and biodegradation properties [42]. The micropatterned structures in fish-scale acellular tissue allowed excellent cell adhesion and the growth of corneal epithelial and stromal cells. Therefore, tilapia scale acellular tissue was prepared under the brand name Biocornea for anterior lamellar keratoplasty. Surface coating of fibronectin, laminin, collagen IV or FNC on the surface of acellular fish scales could further improve its biocompatibility to promote cell proliferation of human corneal endothelial cells (CECs) [44, 109]. Li *et al.* [43] developed contact-lens-like fish-scale acellular tissue membranes through decellularization using NaOH and decalcification using nitric acid and subsequently cross-linked using 1,4-butanediol diglycidyl ether for CECs culture. The results showed that the morphology of CECs cultured on acellular tissue was polygonal and had specific cell junctions similar to those of normal CECs *in vivo*. Their work showed that acellular fish scales could be employed as a substrate for CECs carriers for corneal endothelial transplantation.

The basic ingredients of FSB are type I collagen, elastin and GAG. FBS-derived products are ideal cardiovascular materials because they are rich in elastin. Liu *et al.* [46] reported an acellular biomaterial derived from FSB through decellularization using both chemical method-treatment with SDS and Triton X-100 and enzymatic method-incubation with DNase for small diameter vascular graft and heart valves applications. The results demonstrated that vascular grafts fabricated using FSB acellular tissue equipped excellent anticalcification properties, as well as good physical properties, biocompatibility and hemocompatibility. To match the mechanical properties of human dura mater, Zhang *et al.* [47] cross-linked acellular FSB decellularized with Triton X-100 solution for dura repair. The resultant acellular tissue met the physical and biological requirements of the dural repair material. Inspired by the fact that the thickness of the chub swim bladder approaches that of the human dura mater, and the smooth surface of the bladder may inhibit tissue adhesion, Li *et al.* [69] developed a novel dura mater substitute through the decellularization of FSB. By comparing different decellularization methods, they found that a combination of physical (freeze-thaw cycling) and enzymatic (DNase treatment) methods was the best choice to minimize residual DNA in acellular tissue while maintaining its mechanical properties. In rabbit dura repair experiments, FSB acellular tissue as a dura mater substitute promoted the recovery of defects, while no tissue adhesion or severe inflammation occurred. In addition, decellularized FSB can serve as a collagen-rich scaffold material for the treatment of full-thickness skin wounds. Kumar *et al.* [110] evaluated the effects of cross-linked and non-cross-linked decellularized fish bladder on wound healing in a rabbit skin wound model. After decellularization of the bladder using sodium deoxycholate detergent with stirring, the acellular tissue was cross-linked with 1,4-butanediol diglycidyl ether. The *in vivo* experimental results showed that the cross-linked acellular tissue group produced less IgG in the serum of rabbits than the non-cross-linked acellular tissue group, and wounds treated with acellular tissue had faster re-epithelialization and neovascularization than the sham surgery group. Moreover, the porous structure of acellular FSB makes it suitable as a carrier for cell delivery. The addition of antimicrobial nanomaterials [71] and bone marrow-derived cells [58] can confer new functions to enhance regenerative effects. As one of the most economically important aquaculture fish species in China, sturgeons account for ∼80% of the world’s total production. Sturgeon bone is the main waste generated during the deep processing of sturgeons in fishery production [111]. Since the main component of both acellular sturgeon cartilage and human cartilage is the same type II collagen, Khajavi *et al.* [81] developed a new bioconstruct from acellular sturgeon cartilage by using SDS and DNase-I solutions under stirring. The bioconstruct demonstrated good potential for application in cartilage tissue engineering, as evidenced by its excellent biocompatibility revealed by stem cell adhesion, proliferation and cartilage differentiation characterizations.

Fish, as lower vertebrates, generally have stronger regenerative abilities than mammals do. For example, the heart of an adult zebrafish can completely regenerate and return to its original state after suffering up to a 20% volume loss, whereas the heart of a mammal cannot withstand such damage. Chen *et al.* [25] used a decellularization approach combining physical (freeze-thaw cycles) and enzymatic (DNases/RNases) methods to prepare acellular tissue from zebrafish ventricles. After the administration of zebrafish acellular tissue, significant recovery of heart function was observed in mice with acute myocardial infarction, whereas only slight effects were observed following treatment with acellular tissue from adult mice. Decellularized zebrafish heart offers an alternative strategy for human cardiac tissue regeneration. Similarly, the remarkable regenerative capacity of zebrafish is also manifested in mammalian neural tissue regeneration. Kim *et al.* [72] reported that zebrafish acellular tissue derived from the brain using the same decellularization methods in previous report [25] showed excellent neuronal viability, significant axonal extensions and dense functional network formation in zebrafish acellular tissue scaffolds, comparing to those of mammalian acellular tissue scaffolds.

The diversity and abundance of aquatic animals have driven the development of studies to produce acellular scaffolds from many different aquatic animals, including fish, such as sea urchins [112], planarians [113], jellyfish [114] and squid [115]. Both sea urchins and planarians have attracted considerable attention because of their great regenerative potency, and studies on the ECM from these animals have focused on investigating the role of ECM components in mutable collagen tissues and regenerative biology. Porous acellular scaffolds from jellyfish and squid were produced to mimic the composition of human skin and urinary conduits, respectively, representing great potential for tissue engineering applications. Collectively, the species and tissue diversity of aquatic animals provide possibilities for the preparation of scaffolds with different medical applications.

## Discussion and future perspectives

In the past few decades, the need for autologous and allogeneic tissues has not met the clinical demands. Coupled with advances in decellularization technology, this has catalyzed acellular tissue xenografts derived from numerous terrestrial animals, such as bovine, pig and sheep commercialization and clinically available for a wide range of tissue engineering and regenerative medicine applications. Acellular tissue derived from tissue decellularization attracts intensive attention due to its outstanding advantages for tissue engineering and regenerative medicine applications, which include the following: (i) the removal of immunogenic cellular components alleviates potential adverse immune responses elicited after implantation; (ii) the inherited physical signals from natural ECM can regulate cell–cell and cell–ECM interactions to provide a native-like environment for tissue repair, recovery and regeneration of defective tissue and (iii) the constituents of cytokines, proteins and growth factors (biological signals) inside these decellularized tissues coordinate inflammatory responses, epithelialization and remodeling processes. Based on the excellent properties of acellular tissue stated above, it has a great effect in promoting the regeneration of many tissues, especially bone, cartilage, skin, cornea and cardiovascular tissues. However, decellularized tissue derived from various terrestrial animal tissues may pose risks of transmission of zoonotic pathogens between mammalian species. In addition, residual xenoantigens, including the α-Gal epitopes inherited from terrestrial animals, may remain due to inappropriate decellularization processes, thereby inducing a detrimental immune response after being implanted into the human body. To address this issue, several studies have been conducted to reduce the residual amounts of these antigens. Heart valves decellularized from pigs deficient in α-Gal epitopes still induced mild reactions, implying that decellularization could not sufficiently prevent the immunogenicity of xenogeneic implants [50]. One of the main advantages of using fish tissue to prepare an acellular matrix is the absence of the α-Gal epitopes in fish tissue, which is a major xenoantigen that induces an immune response in xenotransplantation [66]; thus, the use of acellular tissue from non-mammalian species is gaining increasing attention. Decellularized tissues from fish share some similarities in terms of structure and function with mammalian extracellular matrices. Additionally, because of their high collagen content and unique lipid structure, they exhibit good hydrophilicity, sufficient water content and good biocompatibility, which can enhance the metabolic activity and proliferation of cells and support 3D cell growth. Therefore, decellularized fish tissues have potential applications in various areas, such as cardiovascular materials, nerve tissues and healing of complex traumas, such as burns, diabetic ulcers, acute battlefield injuries, bone regeneration and corneal construction.

Aquatic animals, including fish, are more diverse than terrestrial mammals are. Certain species possess unique physical and biological signals that are suitable for specific clinical applications. The development of new regenerative materials is the source of innovation, which will broaden the applications of tissue engineering and regenerative medicine. The sources of decellularized tissues include, but are not limited to, organs (skin, cartilage, scale and bladder) and species (fish, sea urchins, planarians and jellyfish). Decellularized squid mantle tissue was developed as a potential corneal stromal substitute for corneal regeneration owing to its unique advantages, such as being rich in type I collagen and sandwich structure [86]. After decellularization and cleaning using SDS and unobstructed brain imaging cocktails, this corneal stromal substitute was characterized in a rat dorsal muscle transplant for its biocompatibility. The results showed that the corneal stromal substitute was non-cytotoxic and completely degraded after 4 weeks. Moreover, decellularized tissue promoted regeneration of the corneal stroma without inducing severe immune rejection in rabbit corneal interlamellar implantation. In addition, a decellularized marine demosponge-based bone graft was developed through base decellularization and acid demineralization owing to its unique structure and highly porous and interconnected canal systems, which are favorable for cell infiltration, nutrient diffusion and waste removal [116]. The *in vitro* experimental results showed that this bone graft was derived from decellularization using NaOH solution-supported human mesenchymal stromal cell attachment, proliferation and differentiation (chondrogenic, adipogenic and osteogenic). Tissues of some aquatic animals are equipped with special structures and ingredients. In clinical practice, it is imperative to develop an advanced wound dressing that can prevent initial bacterial adhesion to the wound area. The micro topographical structures of shark skin endow it with antifouling and self-cleaning properties. This is primarily because (i) their surface texture size is small, resulting in fewer contact points for bacterial attachment (attachment point theory), and (ii) the microstructure on the surface provides a physical barrier, thus preventing bacteria from forming small colonies (number of distinct topographic features) [117]. If the microstructure of shark skin tissue can be maintained during decellularization, wound dressings made from shark skin tissue will inherently resist bacterial invasion. Similarly, the ingredients in Atlantic cod skin-omega-3 fatty acids enable acellular tissue-Kerecis Omega3 wound accelerating wound recovery by regulating inflammation and vascularization in the wound area [95]. The above studies show that certain aquatic animal tissues have unique advantages. Although the macrostructures of various tissues are similar, their microstructures differ, as does the abundance and types of proteins they contain. This uniqueness in tissue type enables them to play important roles in guiding cell fate [118].Therefore, the effects of acellular tissues from different organs and species on cell behavior and tissue regeneration require further investigation. Although not all aquatic animals possess the best mechanical characteristics, they can still provide the desired shape and mechanical strength through cross-linking, gel formation and 3D bioprinting. Furthermore, pure acellular tissue may not be sufficient to meet clinical needs, and combining nanoparticles or growth factors, various cytokines and trace elements may become a future trend [119].

Ideally, decellularization will remove all cellular components in the tissue that may trigger a deleterious immune response in the host receptor while preserving the original structure, mechanical integrity and biological activity of the ECM. However, current decellularization processes inevitably alter the natural structure and composition of the ECM to some extent [50]. The biggest challenge in decellularization is achieving the optimal method to minimize ECM disruption while maximizing the removal of cellular components from the tissue. Because the efficiency of removing cellular components from the tissue also depends on the type, thickness and density of the tissue, there is no ‘gold standard’ method for decellularization. Decellularization methods include physical, chemical and enzymatic methods, each with its own advantages and limitations (Table 1). The common disadvantages of these decellularization methods are long processing times, irreversible ECM structural damage and toxicity to host cells caused by the residual chemical components and enzymes used in decellularization processes. For fish tissues, decellularization with supercritical fluid using inert substances, such as carbon dioxide, as detergents will show significant advantages. The supercritical carbon dioxide (SCCO_2_) process has been used for the decellularization of terrestrial animal tissues but has not yet been used for fish tissues. Carbon dioxide has a low viscosity and high delivery characteristics. Similar to the principle of critical point drying, carbon dioxide flows at a controlled rate, removing cellular components from the tissue with minimal damage to the mechanical properties of the tissue ECM. In addition to the chemical-free nature of the treatment process and the short processing time, the resultant decellularized tissue is dry, which is beneficial for the long-term storage of the tissue, avoiding the need for freeze-drying. SCCO_2_ has been used for the decellularization of porcine corneas and aortas. Huang *et al.* [120] prepared a new decellularized scaffold for corneal tissue engineering using SCCO_2_ technology. They decellularized porcine corneas in 60% ethanol for 80 min at 350 bar and 45°C. The results showed that the residual DNA content in decellularized corneas was significantly reduced, indicating that the cellular components were effectively removed. Chen *et al.* [121] used SCCO_2_ technology to prepare a novel decellularized cartilage scaffold from porcine articular cartilage and used it as a scaffold to treat articular cartilage defects. The operations of the SCCO_2_ were immersed in 75% ethanol at 200–350 bar and 30–50°C for 40 min. DNA quantification analysis revealed that the residual DNA content in the decellularized cartilage met the requirements for DNA content in decellularized tissue for medical implants. It is rational to predict that the SCCO_2_ decellularization method can also meet the requirements for decellularizing fish tissues after optimizing the process parameters.

**Figure 5.**
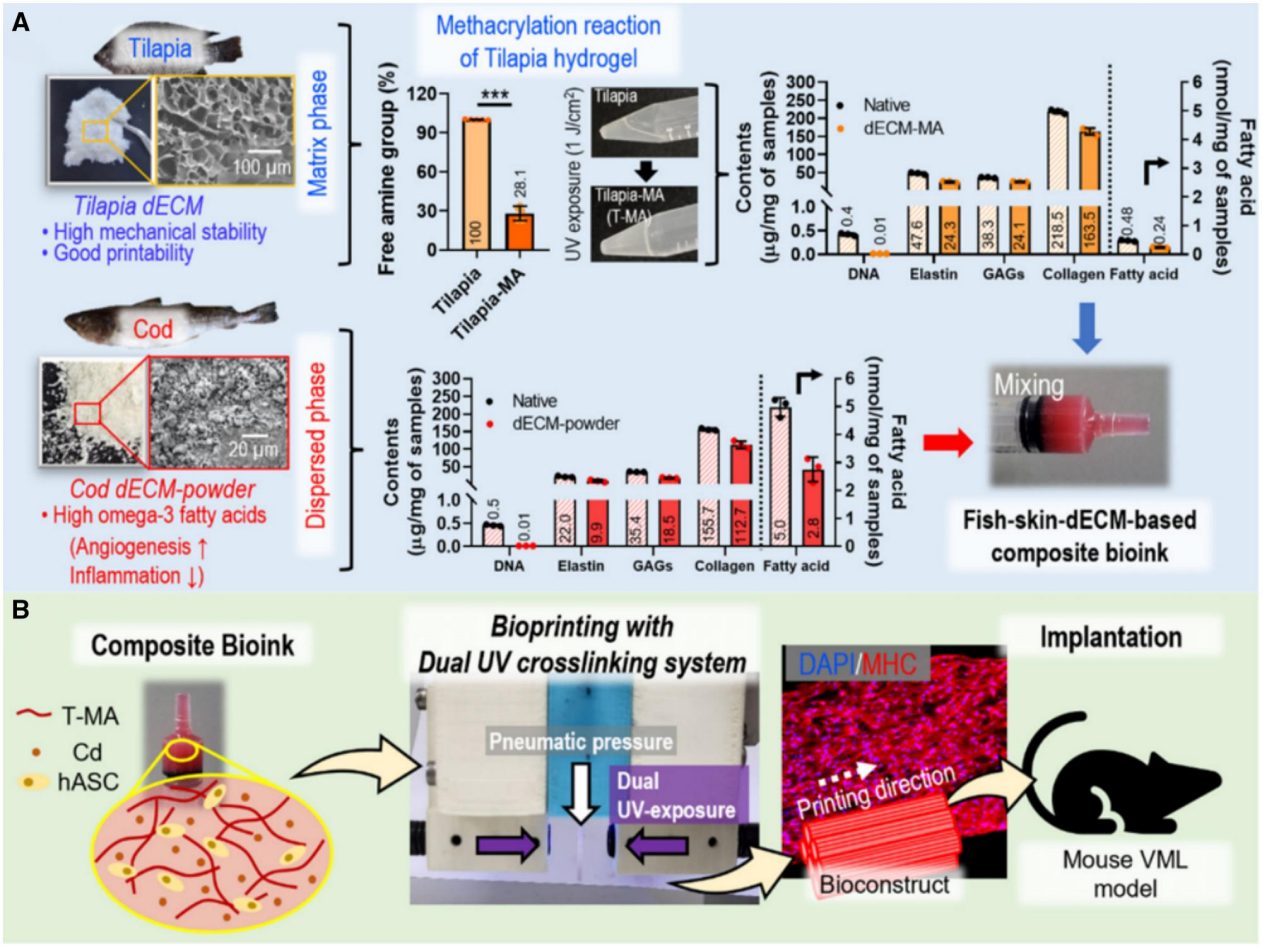
Schematic diagram of the process of cellular fish skin bioconstruct characterization, fabrication and validation in a mouse volume muscle loss model. Reproduced from Ref. [68] with permission from AIP Publishing @ 2024.

Another important application of the acellular tissue of fish is the used as 3D printing bio-ink materials. 3D printing provides a fast way to construct 3D scaffolds for cell growth and tissue regeneration with precisely controlled microstructures (porosity, pore size, etc) that enable customization according to the geometry of the patient’s defect area [122]. This groundbreaking development provides a new approach for creating functional tissues and organs, addressing key challenges in tissue engineering and regenerative medicine. Bio-ink materials are used in 3D bioprinting to fabricate tissue structures. They provide not only the necessary structural support for 3D cell structures to ensure their stability and integrity but also some bioactive signals to enhance biocompatibility of tissue constructs. One of the main sources of bio-ink materials commonly used in 3D bioprinting is synthetic polymers, such as polylactic acid and polycaprolactone (PCL) [123]. They provide the advantage of controllable and repeatable manufacturing, thus allowing the printed scaffolds to exhibit highly consistent mechanical properties, degradation rates, structure and surface morphology. Natural polymers, such as collagen, chitosan and hyaluronic acid, are also sources of bio-ink materials. Owing to their biological compositions that promote cell adhesion, growth and differentiation, these natural polymers render printed scaffolds with excellent bioactivity. To achieve better cell growth in scaffolds, it is a rational strategy to mix synthetic and natural polymers together and print them to form anatomical porous scaffolds that have the advantages of both synthetic and natural polymers. Pan *et al.* [124] reported a new type of bone scaffold manufactured using 3D bioprinting with decellularized bone from α-Gal epitopes-deficient pigs and PCL. The results showed printed scaffold equipped the advantages of synthetic polymers, such as the consistency of the size and porosity of interconnected pores within each scaffold, and excellent mechanical properties, with the benefits of natural polymers, such as the promotion of cell growth and osteogenic differentiation. Recently, Jo *et al.* [68] developed a new fish skin acellular tissue bio-ink for 3D bioprinting, as shown in Figure 5. In addition to human adipose-derived stem cells, this bio-ink also includes acellular skin tissue from cod and tilapia. Cod fish skin acellular tissue as a dispersed phase provided omega-3 fatty acids, which could induce wound angiogenesis and inhibit inflammation, whereas tilapia skin acellular tissue served as a matrix phase to offer high mechanical stability and good printability. The decellularization methods included physical treatment (freeze-thaw cycles) and chemical treatments (incubation with Triton X-100). Under ultraviolet irradiation, the bioconstructs formed by the methacrylated acellular tissue of tilapia skin were filled with cells and contained various bioactive components inherent from cod skin (omega-3 fatty acids). After implantation into a mouse volume muscle loss model, these constructs printed with the cell-laden bio-ink demonstrated significant promotion of muscle tissue regeneration. Aquatic animals, which are abundant and more diverse than terrestrial animals, will provide sufficient sources for the preparation of bio-inks from ECM bases for various tissue regeneration applications, thereby ensuring the supply of 3D bioprinted tissue scaffolds for various clinical applications.

Downstream processes after decellularization, such as cross-linking and surface modification, have been proven effective in improving the physical and biological properties of decellularized tissues [119, 125]. Cross-linking is a method in which one polymer chain is connected to another, typically by a covalent or ionic bond, to enhance the properties of materials. The stability of the ECM structure during decellularization can be affected to varying degrees, leading to adverse effects such as rapid degradation and poor mechanical properties after transplantation into the body. Moreover, the removal of cellular components does not completely eliminate the immunogenicity of decellularized tissue. Therefore, cross-linking treatment between or within collagen fibers in acellular tissue is beneficial for further reducing problematic immune responses and improving the mechanical strength, thermal stability and structural stability of decellularized tissue, further enhancing the degradation properties and biocompatibility of decellularized tissue.

## Conclusion

In this review, we provide an overview of the current research status on fish tissue decellularization, including various methods for preparing fish-derived decellularized tissues, mechanisms, advantages and disadvantages. We enumerated representative studies and described their decellularization processes. Applications of acellular tissues from different fish tissues prepared by specific decellularization methods in tissue engineering have also been exemplified. Based on the unique physical and biological signals of different fish tissues, specific applications of decellularized tissues in tissue engineering and regenerative medicine have been elucidated. The application of fish acellular tissues in tissue engineering and regenerative medicine is an emerging field of research. Further exploration of the optimal decellularization strategies to prepare effective acellular tissues with no chemical and immunogenic cellular residues, expanding aquatic species based on clinical customization needs, and collaborating with advanced 3D bioprinting technology will advance the development of innovation biomaterials for tissue engineering and regenerative medicine, and the immense clinical potential of decellularized aquatic animal tissues will be fully realized.
